# Editorial: Biology, systematics, and evolution of ferns and lycophytes in the omics era, volume II

**DOI:** 10.3389/fpls.2025.1629348

**Published:** 2025-07-15

**Authors:** Li-Yaung Kuo, Alexandre Salino, Thaís Elias Almeida

**Affiliations:** ^1^ Institute of Molecular & Cellular Biology, National Tsing Hua University, Hsinchu, Taiwan; ^2^ Programa de Pós-Graduação em Biologia Vegetal, Universidade Federal de Minas Gerais, Belo Horizonte, MG, Brazil; ^3^ Universidade Federal de Pernambuco, Departamento de Botânica, Centro de Biociências, Recife, PE, Brazil

**Keywords:** allopolyploidy, ferns, lycophytes, multi-omics, organellar phylogenomics, RNA editing

Ferns and lycophytes represent the earliest-diverging lineages among extant vascular plants and occupy critical positions in bridging knowledge gaps of land plant evolution (Pryer et al., 2001; Marchant et al., 2022). However, genomic research and understanding of these groups remain limited, largely due to their enormous genome sizes and limited economic interest (Kuo and Li, 2019). Partly as a result, model-organism-scale surveys in ferns and lycophytes have progressed slowly, delaying broader biological insights (Marchant et al., 2022). Nonetheless, recent advances in sequencing technologies have boosted the generation of phylogenomics, transcriptomics, and metagenomics data, among others, which are increasingly integrated into multi-omics approaches. These developments are helping to overcome the traditional limitations of working with non-model plants like most ferns and lycophytes, significantly advancing our understanding of their evolutionary biology. Through such multi-omics approaches, genomics and expression profiles of ferns and lycophytes can now be readily generated to investigate their biological processes in different tissues, developmental stages, and responses to various biotic/abiotic factors (e.g., Li et al., 2018, 2023; Peng et al., 2024). This enables us to study the unique and intriguing aspects of fern and lycophyte evolution, further clarifying both known and unresolved questions about land plant evolution, as covered by the articles in this Research Topic.

Allopolyploidy, a prevalent mode of speciation in ferns and one that may occur more frequently in these plants compared to their sister lineages (Wood et al., 2009; Barker et al., 2016), offers unique insights into genome evolution. *Phegopteris decursive-pinnata* Fée (Thelypteridaceae), an allotetraploid fern species derived from diploid ancestors of *P. koreana* B.Y.Sun & C.H.Kim and *P. taiwaniana* T.Fujiw., along with their artificially produced diploid F1 hybrids, has served as the model by Katayama et al. to investigate subgenome dominance at the transcriptional level. Comparative transcriptomics revealed homeolog expression bias in some genes, with a slight bias favoring the *taiwaniana*-subgenome dominance. Furthermore, comparisons between the diploid F1 hybrids and the naturally evolving allotetraploid suggest that the observed expression differences in the latter might have emerged during its evolutionary establishment (Katayama et al.). These significant findings highlight that, along the allopolyploid speciation pathway, heterozygosity in ferns can become not only stably fixed at the genomic level but also strongly maintained at the level of gene expression.

RNA editing is an intriguing aspect of molecular evolution that commonly occurs in the organellar genomes of ferns and lycophytes (Takenaka et al., 2013; Fauskee et al., 2025). Their mitochondrial and plastid transcripts exhibit some of the highest known levels of RNA editing among land plants, including frequent C-to-U and U-to-C post-transcriptional modifications. These RNA edits occur predominantly in protein-coding sequences (CDS), where they can restore the function of essential genes, effectively acting as correctors of underlying DNA mutations. In *Isoetes*, an aquatic lycophyte lineage, Pereira et al. assembled organellar genomes and transcriptomes from multiple species. The authors compared genetic divergence within the genus by examining both DNA polymorphisms and post-transcriptional variation, i.e., at the RNA and amino acid levels. Their findings revealed a notable degree of evolutionary conservatism in RNA edits dating back to the deepest divergences of extant species. At the amino acid level, these edits often increased the hydrophobicity of organellar proteins. Also, RNA editing introduces additional genetic diversity into the transcriptome, particularly evident in mitochondrial CDS genes.

Organellar phylogenomics has also proven instrumental in clarifying the evolutionary relationships of the enigmatic plant lineages (Wicke and Schneeweiss, 2015). By assembling complete plastomes and mitogenomes, Kuo et al. reconstructed a robust phylogenomic framework encompassing all genera in the fern family Ophioglossaceae, including *Rhizoglossum* C.Presl, which was sampled phylogenetically for the first time. Importantly, this phylogenomic tree of Ophioglossaceae resolved long-standing controversies of relationships at the subfamily level, better aligning with their morphology and genome size evolution. On these phylogenomic bases, the authors further examined putative horizontal and intracellular gene transfers (HGT and IGT) in these organellar genomes. One of their interesting findings is prevalent HGT in Ophioglossaceae mitogenomes, with mtHGTs originating predominantly from mitogenomes of other fern lineages and angiosperm lineages containing root parasites. Phylogenies of MORFFO, Mobile Open Reading Frames in Fern Organelles, in their study also revealed HGT-like or even IGT-like patterns.

Finally, the interplay between lycophytes and their symbiotic fungi is emerging as a vital yet largely unexplored subject in the coevolution of land plants. Lin et al. applied metabarcoding strategies to investigate the phyllosphere mycobiome across various aboveground tissues of the Lycopodiaceae species *Huperzia asiatica* (Ching) N.Shrestha & X.C.Zhang, which produces significant amounts of Huperzine A (HupA), a pharmacologically important alkaloid that may require co-synthesis with fungal partners. For comparison, the mycobiome of a non-HupA-producing Lycopodiaceae species, *Diphasiastrum complanatum* (L.) Holub, was also analyzed. Their findings, which contrasted HupA concentrations across tissues with the corresponding mycobiomes, reveal that members of certain fungal genera are closely associated with HupA biosynthesis. This study underscores the potential of lycophyte–fungus interactions and their symbiotic coevolution in producing secondary metabolites. Importantly, the discovery of such frontier natural products can be greatly facilitated by integrative, multi-omics approaches.

Together, these studies collectively advance our understanding of fern and lycophyte biology, systematics, and evolution across omics data. They reveal unique evolutionary processes in ferns and lycophytes and offer new frameworks for studying plant diversification, genomic signatures, and symbiosis. As foundational members of the vascular plant lineage, these groups hold untapped potential for answering long-standing evolutionary questions and exploring novel biochemical pathways.
